# Social networks in relation to self-reported symptomatic infections in individuals aged 40–75 - the Maastricht study –

**DOI:** 10.1186/s12879-018-3197-3

**Published:** 2018-07-04

**Authors:** Stephanie Brinkhues, Miranda T. Schram, Christian J. P. A. Hoebe, Mirjam E. E. Kretzschmar, Annemarie Koster, Pieter C. Dagnelie, Simone J. S. Sep, Sander M. J. van Kuijk, Paul H. M. Savelkoul, Nicole H. T. M. Dukers-Muijrers

**Affiliations:** 1 Department of Medical Microbiology, Maastricht University Medical Centre (MUMC+); CAPHRI, Care and Public Health Research Institute, Maastricht University, P.O. Box 616, 6200 MD Maastricht, The Netherlands; 2 Department of Sexual Health, Infectious Diseases and Environmental Health, Public Health Service South Limburg, Postbus 33, 6400AA Heerlen, The Netherlands; 3 Department of Medicine, Maastricht University Medical Centre (MUMC+); CARIM, Cardiovascular Research Institute Maastricht, Maastricht University, P.O. Box 616, 6200 MD Maastricht, The Netherlands; 4 Centre for Infectious Disease Control, National Institute for Public Health and the Environment (RIVM), Bilthoven, University Medical Centre Utrecht, Julius Centre for Health Sciences and Primary Care, Utrecht, The Netherlands; 5 Department of Social Medicine; CAPHRI, School for Public Health and Primary Care, Maastricht University, P.O. Box 616, 6200 MD Maastricht, The Netherlands; 6 Department of Epidemiology, CARIM, Cardiovascular Research Institute Maastricht; CAPHRI, Care and Public Health Research Institute, Maastricht University, P.O. Box 616, 6200 MD Maastricht, The Netherlands; 7 Department of Clinical Epidemiology and Medical Technology Assessment (KEMTA), Maastricht University Medical Centre (MUMC+), P.O. Box 616, 6200 MD Maastricht, The Netherlands; 8 Department of Medical Microbiology & Infection Control, VU University Medical Center, Amsterdam, The Netherlands

**Keywords:** Social network, Upper respiratory tract infection, Lower respiratory tract infection, Gastrointestinal tract infection, Urinary tract infection, Public health

## Abstract

**Background:**

Most infections are spread through social networks (detrimental effect). However, social networks may also lower infection acquisition (beneficial effect). This study aimed to examine associations between social network parameters and prevalence of self-reported upper and lower respiratory, gastrointestinal and urinary tract infections in a population aged 40–75.

**Methods:**

In this population-based cross-sectional cohort study (*N* = 3004, mean age 60.0 ± 8.2 years, 49% women), infections within the past two months were assessed by self-administered questionnaires. Social network parameters were assessed using a name generator questionnaire. To examine the associated beneficial and detrimental network parameters, univariable and multivariable logistic regression was used.

**Results:**

Participants reported an average of 10 people (alters) with whom they had 231 contacts per half year. Prevalences were 31.1% for upper respiratory, 11.5% for lower respiratory, 12.5% for gastrointestinal, and 5.7% for urinary tract infections. Larger network size, and a higher percentage of alters that were friends or acquaintances were associated with higher odds of upper respiratory, lower respiratory and/or gastrointestinal infections (detrimental). A higher total number of contacts, higher percentages of alters of the same age, and higher percentages of family members/acquaintances were associated with lower odds of upper respiratory, lower respiratory and/or gastrointestinal infections (beneficial).

**Conclusion:**

We identified both detrimental and beneficial associations of social network parameters with the prevalence of infections. Our findings can be used to complement mathematical models on infection spread, as well as to optimize current infectious disease control.

**Electronic supplementary material:**

The online version of this article (10.1186/s12879-018-3197-3) contains supplementary material, which is available to authorized users.

## Background

Social networks are assumed to have a powerful influence on health [1–8]. The social network of an individual can be defined as the web of social relationships that surround that individual (ego), referring to contacts with other individuals (alters) [2]. Social relationships may enhance the ability to resist infection but may also act as a vehicle for the transmission of infections. Cohen et al. (1997) demonstrated that individuals with a larger and/or more diverse social network are less susceptible to upper respiratory tract infections (URIs) [9]. Another study showed that smaller social networks were associated with poorer immune response to influenza vaccination in young and healthy adults [10]. In addition, better immune function has been found to be associated with social support [4]: higher levels of social support showed an association with increased natural killer cell activity and decreased interleukin-4 concentration [11, 12]. However, having large and diverse social networks may not always be protective. Hamrick et al. (2002) found that larger social networks were associated with increased numbers of URIs when host resistance to infectious agents was compromised (e.g. among individuals with high levels of stress) [13]. Large social networks were associated with close proximity interactions with a broad range of alters and hence an increased risk of exposure to a broad range of infectious agents. Infectious agents from an infected person can reach a susceptible alter in close proximity, which is typically within a distance of 3 m or less [14, 15]. Previous studies have shown that close-proximity interactions were highly relevant for infectious disease transmission [14, 16, 17], and that close contacts are a better proxy for several infection transmissions than total contacts [14, 18].

To date, it is not yet fully understood which social network characteristics are related to the risk of infections, whether these characteristics have detrimental or beneficial effects or both, and whether relations differ by type of infection. Most previous research on the transmission of infectious disease through social contacts was done using mathematical disease transmission models [19–27]. Previous studies included measures on degree (which is the total number of alters), mixing patterns (percentages of alters younger than, same age or older than ego/percentages same sex as ego), contact patterns (frequency and duration of contact), relationships (e.g. household member/friend/colleague) and social distance [19–26, 28–34].

Furthermore, most studies on networks and infections so far addressed URIs alone [23, 27, 28, 30, 33, 34]. Also, there is a lack of direct comparative data for different types of infections. Different infectious diseases require different modes of contact for transmission [17, 35]. URIs and lower respiratory tract infections (LRIs) are mainly transmitted via droplets (sneezing, coughing), whereas gastrointestinal infections (GIs) require surface contamination and are related to food, personal hygiene and/or close contact, and urinary tract infections (UTIs) are more likely to arise from self-transmission or compromised immunity [35].

Infectious diseases are a major challenge in health care of the older persons [36], aging is associated to increased susceptibility to infections caused by an age-related comprised immune system [37, 38]. Therefore, insights into risk factors for infections would be highly relevant to inform infectious disease control strategies in middle-aged and older persons.

The current study examines the detrimental as well as the beneficial associations between a broad range of network parameters and URI, LRI, GI, and UTI. Comparison across multiple infections will increase robustness of the findings and provide insight into the social network related determinants of infections in the specific group of people aged 40–75.

The current study adds insights into empirical egocentric social network data with infection prevalences on four different infectious diseases in an epidemiological study. This may contribute to enhancement of current infectious disease control, especially by non-pharmaceutical infection prevention strategies [39, 40]. Moreover, by estimating population social structures directly from egocentric contact data, this article provides new information for the estimation of transmission parameters, and thus a basis for more realistic projections of epidemiological data and the effects of interventions by mathematical disease modelling.

## Methods

### Study population

In the present study, we used data from an observational prospective population-based cohort study. The rationale and methodology of The Maastricht Study have been described by Schram et al. (2014) [41]. All individuals living in the southern part of the Netherlands and aged between 40 and 75 years were eligible for participation. Recruitment strategies have been described previously [41]. We included cross-sectional data from the first 3451 participants (baseline survey between November 2010 and September 2013) [41]. Participants adhere to a protocol that covers 4 half day visits to The Maastricht Study research center [41]. The present study includes data from assessments and questionnaires that are given within the first study site visit. Of the 3451 participants, 3004 individuals provided data on social network and infections. The participants without social network and infection data (*n* = 447, 12.9%) did not differ from those with these data with respect to sex, educational level, smoking status, alcohol use, diabetes status or body mass index (BMI). However, participants who did not provide social network and infection data were slightly younger than those who did (mean age 59 versus 60 years, (*p* < 0.001)).

### Measurements

#### Social network

Multiple previous studies applied various methods and techniques to collect empirical data on social networks and contact patterns, such as reviewed by Read et al. [17].

In the present study, social networks were identified by a name generator, one of the best known and most widely used instruments for examining ego-centered network data [42]. The name generator/interpreter is used to map the ego-centered social network and to collect information about the alters of an ego-centered social network, resulting in a detailed description of a participant’s social network. An ego-centered network is defined as a network centered on a specific individual, called the ego [43]. Each person who has a relationship with an ego was defined as an “alter” [44]. The social network measured within this study mainly focused on close-proximity interactions [14].

A detailed description of the name generator questionnaire can be found elsewhere [45]. In brief, the name generator first requires a respondent to identify actual persons (alters) in response to seven questions on different types of contacts (e.g. persons who advised them on problems or persons they visited for social purposes or that they could go out with sometimes). For all seven types of contacts, they were asked to indicate their frequency of contact with this person over the last six months (daily or weekly, monthly, quarterly, and half-yearly). In total, participants could name a maximum number of 40 alters. Next, several additional questions about all alters named were asked (sex, age, type of relationship, geographical proximity).

Moreover, participants were asked to rate the statements “most of my friends know each other” and “my best friends know my family” on a five-point Likert scale ranging from strongly agree to strongly disagree. Finally, participants had to indicate whether they were a member of a club (yes/no), and, if so, to identify the club(s) concerned (sports club, volunteer organization, religious group, self-support group, discussion group, Internet club, or another organization) and how often they frequented this club (daily/weekly, monthly, occasionally).

#### Self-reported infections

In a structured questionnaire, participants were asked whether they had suffered from sudden symptoms such as a cough, runny nose, sore throat, fever, vomitus with fever, or pain when urinating, in the previous two months. They were also asked whether they had suffered from sudden onset of influenza, pneumonia, urinary tract infection, middle ear infection, diarrhea, or skin infection in the previous two months. All of these questions were yes/no questions.

#### General measurements

Self-administered questionnaires were used to assess educational level (low (no education, primary education, and lower vocational education)/medium (intermediate vocational education, higher secondary education, and vocational education)/high (higher professional education, university)), employment status (employed/retired or not employed/not known), smoking status (never/former/current) and alcohol consumption (non-consumers/low consumers (≤7 glasses per week for women, ≤ 14 glasses per week for men)/high consumers (> 7 glasses per week for women, > 14 glasses per week for men)). To determine type 2 diabetes, all participants (except those who used insulin) underwent a standardized 7-point 75 g. OGTT after an overnight fast. Height, weight and BMI were assessed as described previously [41], and defined according to the WHO classification (normal (BMI < 25), overweight (BMI 25- < 30), and obese (BMI ≥30)).

### Exposure variables: Social network parameters

First, in the literature we identified several social network parameters that had previously been examined in relation to infections. Next, the social network parameters listed below were computed and used in the current study. The majority of social network parameters used in the current study focused on close-proximity interactions as previous studies had shown their importance in infectious disease transmission [14, 18].

#### Network size (degree)

Previous studies identified social network size as determinant for several health outcomes and it is also widely used in mathematical disease transmission models [9, 10, 13, 16, 19, 25, 28, 33, 42, 46]. Therefore, the degree of the social network was defined as the total number of alters mentioned in the questionnaire and was computed as the size of the ego network (network size).

#### Contact frequency

In line with several studies on mathematical modelling of the spread of infectious disease, we also investigated contact frequency [19, 21, 22, 25, 33]. First, we used highest contact frequency (e.g. daily contact) for every alter as an indicator of the actual contact frequency. For example, if participants reported alter 1 as a person they visited for social purposes, with a frequency of “daily or weekly” and also named the same alter as a person who provided practical help if they were sick, with a frequency of “quarterly”, we considered “daily or weekly” as the actual frequency of contact between the ego and the alter. Second, we recoded the answer categories of the questionnaire in an estimated number of contacts per half year. For example, “half-yearly” was assumed to comprise one contact, “quarterly” two contacts, “monthly” 6 contacts and “daily or weekly” 48 contacts [21]. Third, we computed the sum of all contacts per half year as the total contact frequency. In addition, we computed the percentages of alters that the ego had daily/weekly, monthly, quarterly and half-yearly contact with, for example as the number of daily/weekly contacts divided by network size.

#### Geographical proximity

Previous studies included measures on home contacts and distance from home [21, 22, 28, 33]. In the current study, we calculated geographical proximity as the percentage of all alters that were household members, lived within walking distance, lived less than half an hour away by car, lived more than half an hour away by car, and lived further away (e.g. in another country). For example, we calculated the percentage of household members as the number of alters living in the same household divided by network size.

#### Network heterogeneity

In accordance with another study among social networks in the Netherlands, we also computed heterogeneity of age and sex [42]. To assess sex heterogeneity within the ego’s network, we computed the Index of Qualitative Variation (IQV) by Mueller and Schuessler (1961) [47]. This index indicates the probability that two randomly chosen network alters belong to the same category. The statistical formula for the derivation of the IQV can be found in the Additional file 1. In brief, the IQV is defined as the ratio of observed differences divided by maximum possible differences, where “0” represents a fully homogeneous and “1” a fully heterogeneous network [47]. Observed differences were calculated through multiplication of the total number of men in the ego’s network by the total number of women in the ego’s network. We calculated maximum differences as (network size/ 2)^2^ [47]. The IQV was computed as observed differences/maximum possible differences. We defined age heterogeneity of network alters as the standard deviation of the mean age of all alters of the ego [42].

#### Mixing

According to studies on mathematical infectious disease modelling, we calculated mixing parameters for age mixing patterns (whether the ego had contact with younger, same age or older alters) and sex mixing patterns (whether the ego had contact with alters of the same sex or the opposite sex) [22, 28, 33]. To identify age mixing, we calculated the difference between the ego’s age and the alter’s age for every alter named. Next, we computed the percentages of younger (> 15 years and 5 to 15 years younger), same age (±5 years) and older (> 15 years and 5 to 15 years older) alters for each participant. To indicate sex mixing, we calculated the percentage of same-sex alters. For example, for a female participant the number of her female alters was divided by her network size to obtain the percentage of same-sex alters.

#### Type of relationship

The questionnaire also assessed the type of relationship between the ego and the alter. To the best of our knowledge, this is the first study that examines network composition in terms of the type of relationship. To that end, we computed the percentage of alters that were family members, friends, colleagues and acquaintances. For example, we calculated the percentage of family members within the network as the number of family members divided by network size. Whether the ego had a partner was derived from the social network questionnaire and computed as having/not having a partner. A partner was defined as an intimate relationship with another person.

#### Density

We assessed network density in two questions [42, 48], categorizing density scores separately for density of the ego’s friends and density of the ego’s friends and family. Density was defined as the extent to which alters in the network know each other. Density between friends was computed from the statement “most of my friends know each other” (five-point Likert scale ranging from strongly agree to strongly disagree) and density between friends and family was computed from the statement “my best friends know my family”. We used tertiles to compute three equal groups of low density, medium density and high density.

#### Superficial contacts

We included a proxy for more superficial contacts than close-proximity interactions as transmission of infections may also occur via contact with contaminated surfaces or exposure that does not involve conversation or touch [49]. We therefore constructed a variable representing the total number of club memberships (and the number of clubs the ego frequented on a daily or weekly, monthly or occasional basis) as a proxy for superficial contacts.

#### Close proximity interactions

While all of the types of interactions in the name generator suggest close and direct contact, the questions do not explicitly include information on whether an interaction is physical (e.g. kiss or handshake), face-to-face or by phone/internet. Some interactions such as help with jobs around the house or persons they visited for social purposes require close proximity interactions, whereas other interactions such as advice on problems or provision of emotional support may have occurred by telephone/internet.

To assess the proportion of close proximity interactions, we additionally computed the network size and total contact frequency from those type of interactions with alters that are by definition in close proximity; persons who could offer them practical help if they were sick, persons who helped them with small and larger jobs around the house, persons who were also important for them because of mutual activities, and/or household contacts. The additionally computed social network size of close proximity interactions represented 86% of the total social network size (8,5 alters in a network of 10 alters), and the total contact frequency of close proximity interactions represented 87% of the total contact frequency (202 contacts per half year in a total number of 231 contacts per half year).

In all analyses, the total network size and total contact frequency per half year were used.

### Outcome variables: Self-reported symptomatic infections over the past two months

The symptoms “runny nose” and “sore throat” were pooled as indicators of URI. Influenza, pneumonia and fever were pooled as indicators of LRI. Pain when urinating and urinary tract infection were pooled as UTI. Vomitus with fever and diarrhea were pooled as GI. We excluded cough from the analysis because it is strongly related to smoking and asthma [50], and not exclusively a specific indicator for infection.

**Fig. 1:**
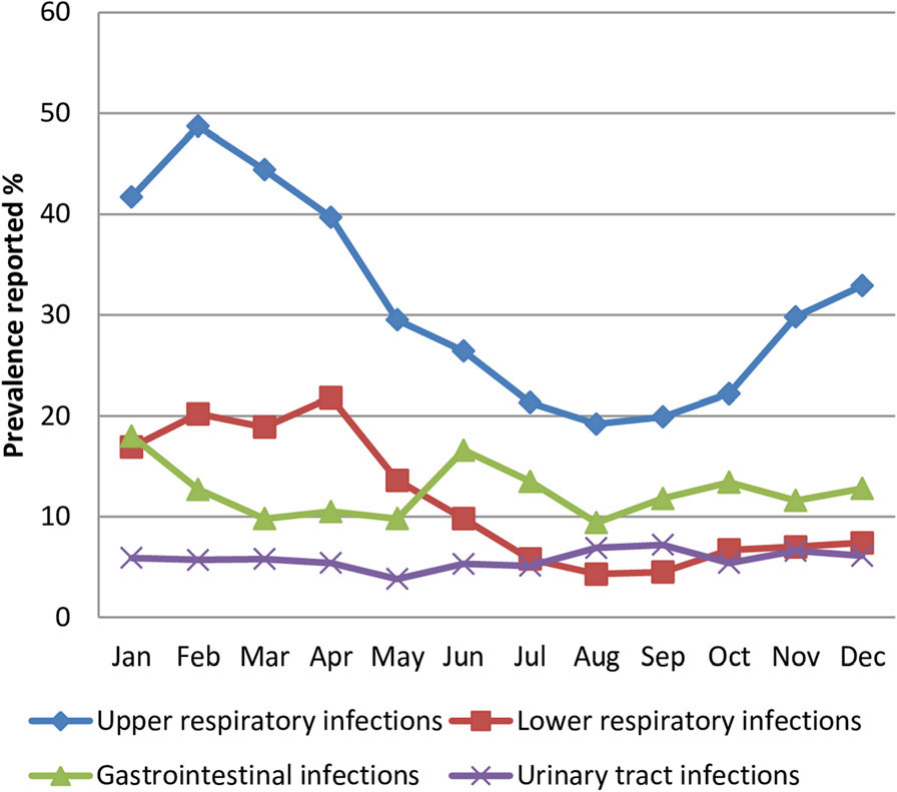
The observed prevalences of URI, LRI, GI and UTI for each month of the year

The observed prevalences for each month of the year (Fig. 1) display the expected seasonality of the diseases.

### Statistical analyses

We performed descriptive analyses to examine the characteristics of the participants in terms of baseline characteristics, prevalence of self-reported infections, and network parameters.

First, we conducted bivariate correlation analyses to rule out multicollinearity between the network variables. With all correlation cut-off values below 0.7, none of the variables were considered collinear.

Second, we conducted univariable logistic regression analyses to assess the association between the exposure variables, i.e. network parameters, and the outcomes of URI, LRI, GI, and UTI. All network parameters were continuous variables, except for density. For every network parameter, odds ratios (ORs) and 95% confidence intervals (95%CI) were calculated.

Network size as (unadjusted) determinant for the four infections was visually presented using polynomials (cubic function).

Third, we built two multivariable models to determine the most important detrimental and beneficial network parameters. We forced network size and total contact frequency into the models as these variables are considered essential for the assessment of detrimental and beneficial social network effects, and have been shown to be related to the transmission of infections as well as to decrease susceptibility to infections [9, 10, 13, 19, 21, 22, 28, 29, 33, 42]. In the detrimental exposure model, we further included all variables that were positively associated with URI (odds ratio > 1), regardless of their statistical significance. In the beneficial exposure model, we further included all variables that were negatively related with URI (odds ratio < 1), again regardless of their statistical significance. Next to the social network size and total contact frequency, we used several social network parameters in percentages, to be able to assess the effect of the composition of the social network independent of the social network size and total contact frequency. For those social network parameters that were computed as percentages within the network, the associations were presented in steps of 10%. Based on an average network size of 10 network members, a change in one network member corresponds to 10%. For the detrimental and beneficial models, we used the stepwise backward method (*p* < 0.1) to obtain the final model, including possible confounders, network size, and total contact frequency. These analyses were repeated for LRI, GI and UTI. We used the variance inflation factor (VIF) to measure collinearity in all regression models. Values for VIF and tolerance did not indicate multicollinearity problems with cut-off values of VIF < 10 and tolerance (1/VIF) > 0.1.

We adjusted all associations for possible confounders, i.e. diabetes status (type 2 diabetes oversampled by design), age, sex, BMI, smoking status, alcohol consumption, educational level, and employment status. We also adjusted all associations for the season in which the measurement took place to account for the likelihood of encountering an infected source. In the multivariable models, associations with *p* < 0.05 were considered statistically significant.

In addition, we tested statistical interaction (effect modification) of the network parameters with sex and age to check whether the associations between network parameters and outcome differed by sex and age. However, none of the interactions of the network parameters with sex and age were statistically significant (*p* > 0.1).

We performed sensitivity analyses to verify the model building process; we replicated multivariable logistic regression analyses by using the complete model instead of backward elimination, and used degrees instead of percentages. The findings were in line with the results presented.

All analyses were conducted using IBM SPSS Statistics version 21.0 (IBM Corp. Armonk, NY, USA).

## Results

The overall study population consisted of 3004 participants with a mean age of 60.0 years. Participants were mainly Caucasian (98.6%), slightly less than half were women (49%), nearly one third had type 2 diabetes (by design), two thirds were overweight (or obese), and one in seven were current smokers (Table 1).

**Table 1:** Characteristics of the participants and prevalences of infections

Individual characteristics	% (*n* = 3004) or Mean (SD)
Sex	
- Men	51.2 (*n* = 1537)
- Women	48.8 (*n* = 1467)
Age	60.0 (8.2)
Educational level	
- Low	33.4 (*n* = 980)
- Medium	28.1 (*n* = 823)
- High	38.5 (*n* = 1129)
Employment status	
- Employed	39.0 (*n* = 1172)
- Unemployed/ retired	51.0 (*n* = 1531)
- Not known	10.0 (*n* = 301)
Diabetes status	
- No type 2 diabetes	71.3 (*n* = 2141)
- Type 2 diabetes	28.7 (*n* = 863)
Body Mass Index	
- Normal (< 25 kg/m^2^)	35.6 (*n* = 1067)
- Overweight (25–30 kg/m^2^)	42.3 (*n* = 1268)
- Obese (≥30 kg/m^2^)	22.2 (*n* = 666)
Alcohol consumption	
- Non-consumers (no alcohol use)	18.3 (*n* = 537)
- Low consumers ((≤7 glasses per week for women; ≤ 14 glasses per week for men)	56.0 (*n* = 1645)
- High consumers (> 7 glasses per week for women; > 14 glasses per week for men)	25.7 (*n* = 755)
Smoking status	
- Never	34.6 (*n* = 1020)
- Former	52.0 (*n* = 1529)
- Current	13.4 (*n* = 394)
Infections (self-reported, past 2 months)	
- Upper respiratory infection (URI)	31.1 (*n* = 921)
- Lower respiratory infection (LRI)	11.5 (*n* = 339)
- Urinary tract infection (UTI)	5.7 (*n* = 170)
- Gastrointestinal tract infection (GI)	12.5 (*n* = 370)
Month of assessment	
- May–October	52.7 (*n* = 1584)
- November–April	47.3 (*n* = 1420)

URI, LRI, UTI and GI in the past two months were reported by 31.1, 11.5, 5.7 and 12.5% of the participants, respectively (Table 1). The prevalences of URI, LRI, GI and UTI for every month of the year were presented in Fig. 1.

On average, participants reported a network size of 10 alters, and 231 contacts per half year (Table 2). Only 15 (0.5%) participants reported a network size of 25 or more alters, and only 10 (0.3%) participants reported more than 750 contacts per half year.

**Table 2:** Means and prevalences of social network parameters

	Mean (SD) or % (n)
Network size (number of alters) (mean, SD)	9.83 (5.17)
Contact frequency	
Total contacts per half year (mean, SD)	231.18 (141.16)
Percentage of alters with daily-weekly ego/alter contact (mean, SD)	48.96 (25.88)
Percentage of alters with monthly ego/alter contact (mean, SD)	23.34 (19.49)
Percentage of alters with quarterly ego/alter contact (mean, SD)	12.50 (15.15)
Percentage of alters with half-yearly ego/alter contact (mean, SD)	15.17 (19.47)
Proximity	
Percentage of alters who are household members (mean, SD)	15.43 (15.08)
Percentage of alters living within walking distance (mean, SD)	28.80 (22.58)
Percentage of alters living less than 1/2 h away by car (mean, SD)	38.35 (24.06)
Percentage of alters living more than 1/2 h away by car (mean, SD)	13.93 (17.80)
Percentage of alters living further away (mean, SD)	3.50 (8.97)
Mixing	
Percentage of same-sex alters (mean, SD)	58.15 (17.72)
Percentage of same-age alters (±5 years) (mean, SD)	44.28 (21.20)
Percentage of younger alters (> 15 years younger) (mean, SD)	6.50 (10.82)
Percentage of younger alters (5 to 15 years younger) (mean, SD)	11.39 (14.29)
Percentage of older alters (> 15 years older) (mean, SD)	26.73 (20.46)
Percentage of older alters (5 to 15 years older) (mean, SD)	12.83 (15.56)
Heterogeneity	
Sex heterogeneity (IQV, range 0–1) (mean, SD)	0.85 (0.21)
Age heterogeneity (SD) (mean, SD)	14.44 (5.17)
Type of relationship	
Participants who have a partner (%)	81.1 (*n* = 2436)
Percentage of alters who are family members (mean, SD)	58.86 (24.03)
Percentage of alters who are friends (mean, SD)	27.16 (21.23)
Percentage of alters who are acquaintances (colleague, neighbor, club mate, other) (mean, SD)	13.98 (16.91)
Proxy for superficial contacts	
Number of club memberships (mean, SD)	1.07 (1.01)
Number of clubs with daily/weekly participation (mean, SD)	0.77 (0.80)
Number of clubs with monthly participation (mean, SD)	0.19 (0.45)
Number of clubs with less frequent participation (mean, SD)	0.10 (0.34)
Network density	
Density friends	
- low density (1–3) (%)	17.3 (*n* = 514)
- medium density (4) (%)	42.9 (*n* = 1273)
- high density (5) (%)	39.8 (*n* = 1179)
Density friends and family	
- low density (1–3) (%)	25.4 (*n* = 753)
- medium density (4) (%)	43.9 (*n* = 1302)
- high density (5) (%)	30.7 (*n* = 912)

**Fig. 2:**
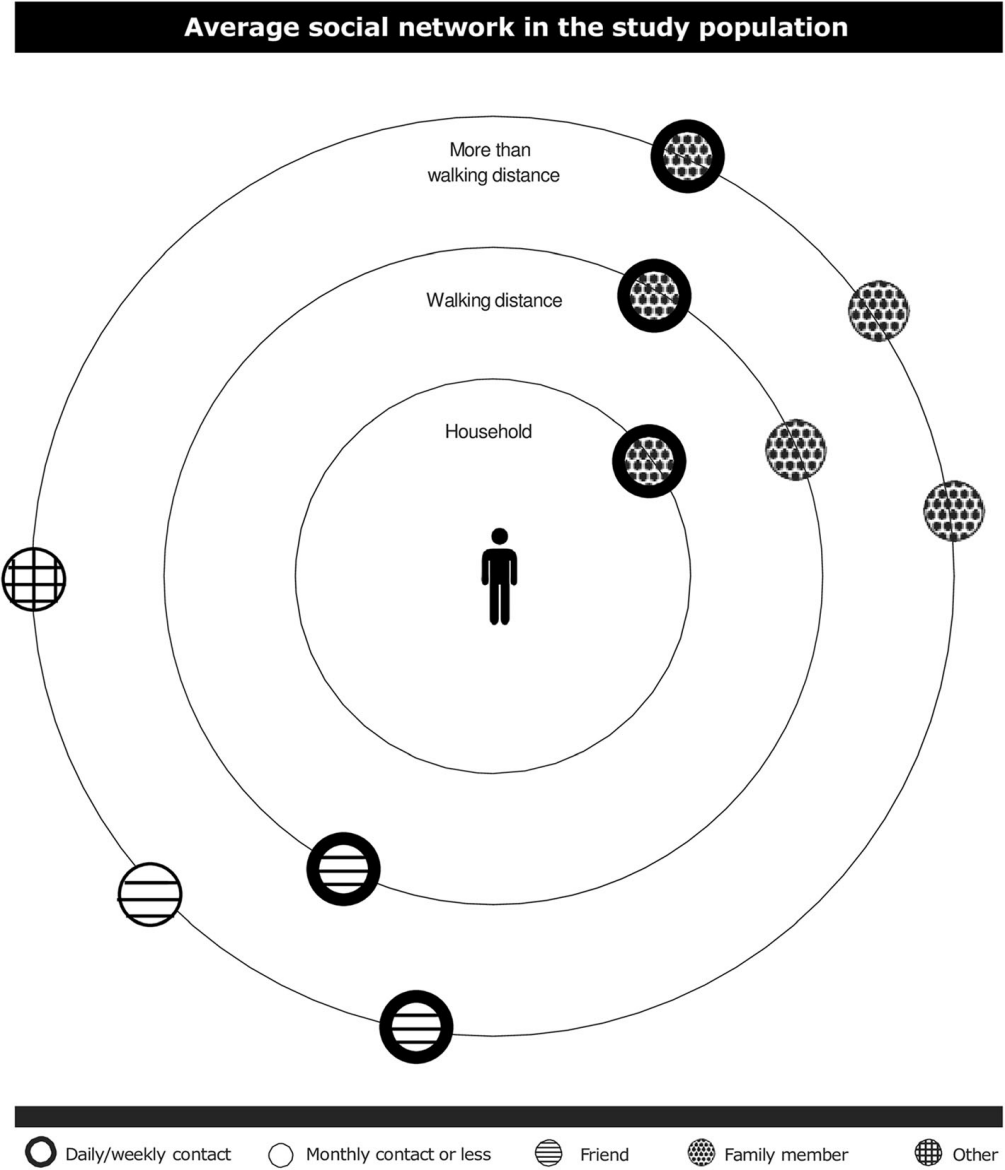
Visualization of the average social network of the study population with regard to composition of contact frequency, proximity, and type of relationship

**Fig. 3:**
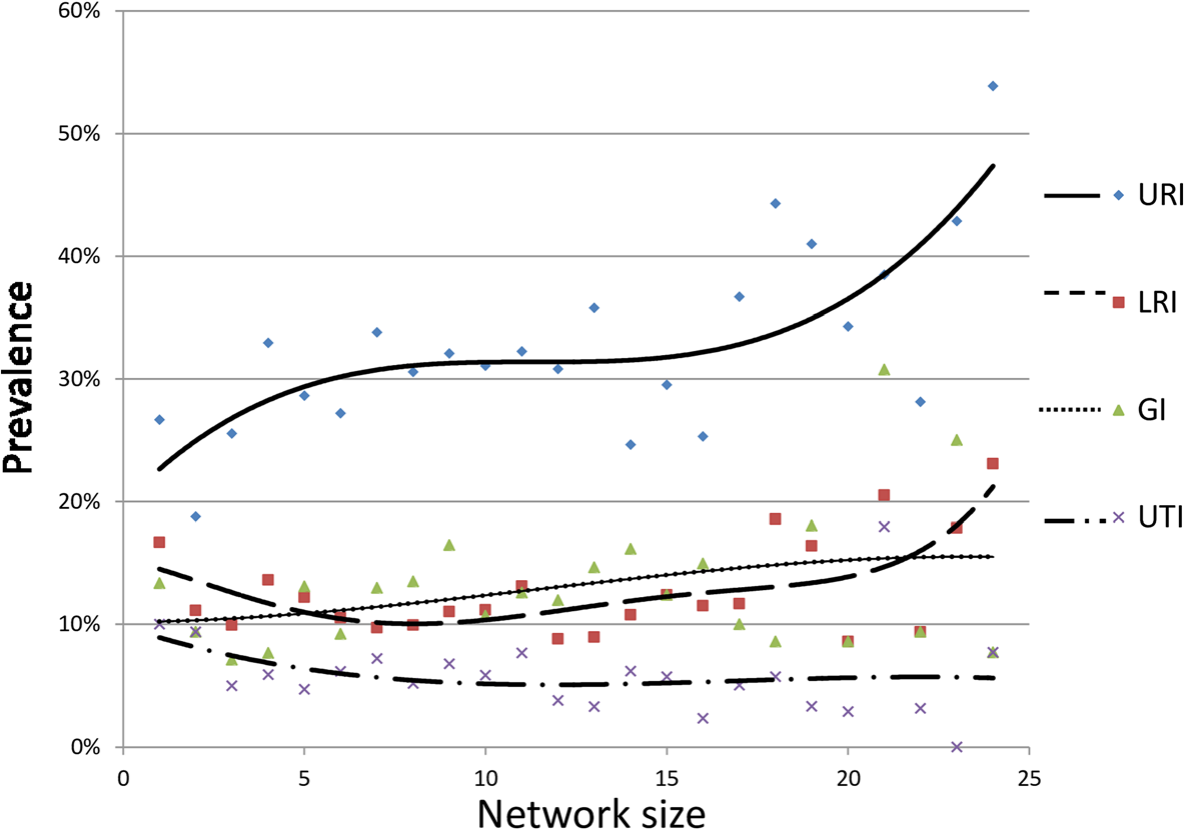
Unadjusted prevalences of URI, LRI, GI, and UTI for network size presented using polynomials (cubic function)

Participants reported to have daily or weekly contact with nearly half the number of their alters, 15% of the alters were household members, and sex heterogeneity in the network was high. About half of the alters were not of the same sex and age (±5 years) as the participant. The majority of participants reported to have a partner. More than half of the alters were family members, one third were friends and the remaining alters were acquaintances such as neighbors, club mates and colleagues (Table 2). Fig 2 visualizes the average social network of the participants, indicating the average network size, with frequency of contacts, the type of relationship, and the proximity of alters. Further, the unadjusted prevalences for URI. LRI, GI and UTI for network size were presented in Fig. 3.

Nearly half of participants reported high density between friends (“my friends know each other”), whereas one third reported a high density between friends and family (“best friends know my family”). On average, participants were a member of one club (Table 2).

The associations of demographic characteristics (sex, age, type 2 diabetes, body mass index, season, educational level, smoking status, alcohol consumption, and employment status) with URI, LRI, GI and UTI were shown in an additional table (see Additional file 2).

### Social network related to URI

Table 3 presents the results of the adjusted univariable analyses, all associations were adjusted for potential confounders. Each additional alter reported was associated with a 2% higher odds of reporting URI. Every additional 10% of the network contacted infrequently (once per half year) or living at larger distance was associated with a 6 and 5% higher odds of reporting URI, respectively. Every additional 10% of the network contacted frequently (daily or weekly), household members, and alters of the same age were associated with a 4, 7, and 5% lower odds of URI, respectively.

**Table 3:** Associations between network parameters and infections, derived from logistic regression analysis, adjusted for characteristics of the participant

	Upper respiratory tract infection		Lower respiratory tract infection		Gastrointestinal infection		Urinary tract infection	
	OR	95% CI	OR	95% CI	OR	95% CI	OR	95% CI
Network size (for every additional alter) (mean, SD)	1.02*	1.00–1.04	1.01	0.99–1.04	1.03*	1.01–1.05	0.99	0.96–1.03
Contact frequency								
Total contacts per half year (for every 10 additional contacts) (mean, SD)	1.00	0.99–1.01	1.00	0.99–1.00	1.00	0.99–1.01	1.01	1.00–1.02
Percentage of alters with daily-weekly ego/alter contact (for every additional 10%) (mean, SD)	0.96*	0.93–0.99	0.97	0.92–1.01	0.95*	0.90–0.99	1.05	0.99–1.12
Percentage of alters with monthly ego/alter contact (for every additional 10%) (mean, SD)	1.00	0.96–1.04	0.99	0.93–1.05	1.05	0.99–1.11	1.03	0.95–1.12
Percentage of alters with quarterly ego/alter contact (for every additional 10%) (mean, SD)	1.02	0.96–1.07	1.08#	1.00–1.16	1.10**	1.02–1.18	0.93	0.82–1.04
Percentage of alters with half-yearly ego/alter contact (for every additional 10%) (mean, SD)	1.06**	1.01–1.10	1.02	0.96–1.08	0.99	0.93–1.05	0.92	0.84–1.01
Proximity								
Percentage of alters who are household members (for every additional 10%) (mean, SD)	0.93*	0.88–0.99	0.98	0.90–1.07	0.87**	0.80–0.95	1.01	0.91–1.13
Percentage of alters living within walking distance (for every additional 10%) (mean, SD)	0.99	0.96–1.03	1.01	0.96–1.07	0.99	0.94–1.04	0.99	0.92–1.06
Percentage of alters living less than 1/2 h away by car (for every additional 10%) (mean, SD)	1.00	0.97–1.04	0.97	0.92–1.02	1.02	0.97–1.07	1.01	0.94–1.08
Percentage of alters living more than 1/2 h away by car (for every additional 10%) (mean, SD)	1.05*	1.00–1.10	1.07*	1.00–1.14	1.07*	1.01–1.14	0.96	0.87–1.06
Percentage of alters living further away (for every additional 10%) (mean, SD)	1.03	0.94–1.13	0.92	0.79–1.07	1.02	0.90–1.15	1.09	0.93–1.28
Mixing								
Percentage of same-sex alters (for every additional 10%) (mean, SD)	1.00	0.96–1.05	1.01	0.94–1.08	1.06#	0.99–1.14	0.93	0.85–1.02
Percentage of same-age alters (for every additional 10%) (±5 years) (mean, SD)	0.95*	0.91–0.99	0.92**	0.86–0.97	0.96	0.91–1.01	0.99	0.92–1.07
Percentage of younger alters (for every additional 10%) (> 15 years younger) (mean, SD)	1.00	0.91–1.09	0.97	0.85–1.10	0.97	0.86–1.10	1.03	0.86–1.22
Percentage of younger alters (for every additional 10%) (5-15 years younger) (mean, SD)	1.03	0.97–1.09	1.09*	1.01–1.18	1.09*	1.01–1.17	0.94	0.84–1.06
Percentage of older alters (for every additional 10%) (> 15 years older) (mean, SD)	1.04#	1.00–1.09	1.03	0.97–1.10	0.99	0.93–1.05	1.03	0.95–1.12
Percentage of older alters (for every additional 10%) (5–15 years older) (mean, SD)	1.02	0.97–1.08	1.07#	0.99–1.15	1.05	0.97–1.12	1.02	0.92–1.14
Heterogeneity								
Sex heterogeneity (IQV, range 0–1) (mean, SD)	1.38	0.91–2.10	1.20	0.65–2.22	0.92	0.53–1.58	1.20	0.54–2.68
Age heterogeneity (per SD increase) (SD) (mean, SD)	1.01	1.00–1.03	1.01	0.99–1.04	1.00	0.98–1.02	1.00	0.96–1.03
Type of relationship								
Participants who have a partner (%)	0.99	0.80–1.23	0.75#	0.56–1.00	0.74*	0.56–0.97	1.35	0.87–2.08
Percentage of alters who are family members (for every additional 10%) (mean, SD)	0.97#	0.94–1.00	0.92**	0.88–0.97	0.91***	0.87–0.96	1.04	0.97–1.12
Percentage of alters who are friends (for every additional 10%) (mean, SD)	1.01	0.97–1.05	1.08*	1.02–1.14	1.12***	1.06–1.18	0.99	0.92–1.08
Percentage of alters who are acquaintances (colleague, neighbor, club mate, other) (for every additional 10%) (mean, SD)	1.04#	1.00–1.10	1.04	0.97–1.12	1.00	0.93–1.07	0.93	0.83–1.03
Proxy for superficial contacts								
Number of club memberships (mean, SD)	1.02	0.93–1.11	1.03	0.91–1.16	1.04	0.93–1.17	0.99	0.84–1.16
Number of clubs with daily/weekly participation (mean, SD)	0.99	0.89–1.10	1.00	0.86–1.16	1.02	0.89–1.18	0.97	0.79–1.20
Number of clubs with monthly participation (mean, SD)	1.10	0.92–1.32	1.18	0.92–1.51	1.06	0.83–1.35	1.06	0.74–1.52
Number of clubs with less frequent participation (mean, SD)	1.01	0.80–1.28	0.97	0.68–1.38	1.15	0.85–1.55	0.95	0.58–1.55
Network density								
Density between friends								
- low density (1–3) (%)	1.00		1.00		1.00		1.00	
- medium density (4) (%)	1.13	0.92–1.38	0.87	0.65–1.16	0.94	0.72–1.24	0.90	0.60–1.35
- high density (5) (%)	1.08	0.86–1.35	0.92	0.67–1.25	0.89	0.66–1.20	1.09	0.71–1.67
Density between friends and family								
- low density (1–3) (%)	1.00		1.00		1.00		1.00	
- medium density (4) (%)	1.16	0.92–1.47	0.93	0.67–1.29	1.01	0.74–1.39	1.13	0.70–1.81
- high density (5) (%)	1.27*	1.00–1.61	0.87	0.65–1.26	1.04	0.75–1.43	1.24	0.77–2.00

All analyses were adjusted for: sex, age, smoking status, diabetes status, BMI, alcohol consumption, educational level, employment status, and season

OR Odds Ratio, 95% CI; 95% Confidence Interval, # *p* < 0.1 *p < 0.05 **p < 0.01 ***p < 0.001

Table 4 presents the results of the multivariable analyses, all associations were adjusted for potential confounders. In the multivariable detrimental exposure model, network size remained independently associated with URI; with each additional alter reported, the odds of reporting an URI was 4% higher. In the multivariable beneficial exposure model, it was further shown that every additional 10 contacts within half a year and every additional 10% of the network of the same age were associated with 1 and 6% lower odds of reporting URI, respectively.

**Table 4:** Detrimental exposure model and beneficial exposure model derived from Bstep logistic regression analyses, adjusted for characteristics of the participant

	Upper respiratory tract infection		Lower respiratory tract infection		Gastrointestinal infection		Urinary tract infection	
	OR	95% CI	OR	95% CI	OR	95% CI	OR	95% CI
Detrimental exposure model								
Network size (for every additional alter)	1.04**	1.01–1.06	1.02	0.99–1.06	1.03#	1.00–1.06	0.96	0.92–1.01
Total contacts per half year (for every additional 10 contacts)	0.99*	0.98–1.00	0.99*	0.98–1.00	1.00	0.99–1.01	1.02*	1.00–1.03
Percentage of alters living more than 1/2 h away by car (for every additional 10%)	N/A		N/A		1.06#	1.00–1.13	N/A	
Percentage of older alters (> 15 years older) (for every additional 10%)	1.05#	0.98–1.09	1.08#	0.99–1.17	N/A		N/A	
Percentage of older alters (5–15 years older) (for every additional 10%)	N/A		1.12*	1.02–1.22	N/A		N/A	
Percentage of younger alters (5–15 years younger) (for every additional 10%)	N/A		1.11*	2.01–1.21	1.08#	0.99–1.16	N/A	
Age heterogeneity (SD)	N/A		1.03**	1.00–1.06	N/A		N/A	
Percentage of alters who are friends (for every additional 10%)	N/A		1.12***	1.04–1.20	1.11***	1.05–1.16	N/A	
Percentage of alters who are acquaintances (colleague, neighbor, club mate, other) (for every additional 10%)	N/A		1.08#	0.99–1.17	N/A		N/A	
Beneficial exposure model								
Network size (for every additional alter)	1.03**	1.01–1.06	1.02	0.99–1.06	1.03*	1.00–1.06	0.96	0.92–1.01
Total contacts per half year (for every additional 10 contacts)	0.99*	0.98–1.00	0.99*	0.98–1.00	0.99	0.98–1.00	1.02*	1.00–1.03
Percentage of same-age alters (± 5 years) (for every additional 10%)	0.94**	0.90–0.98	0.89***	0.84–0.95	0.93*	0.88–0.98	N/A	
Percentage of alters who are family members (for every additional 10%)	0.97#	0.93–1.01	0.91***	0.87–0.96	0.89***	0.84–0.94	N/A	
Percentage of alters who are acquaintances (colleague, neighbor, club mate, other) (for every additional 10%)	N/A		N/A		0.91*	0.84–0.98	N/A	

All analyses were adjusted for: sex, age, smoking status, diabetes status, BMI, alcohol consumption, educational level, employment status and season. OR Odds Ratio, 95% CI; 95% Confidence Interval, # *p* < 0.1 *p < 0.05 **p < 0.01 ***p < 0.001

### Social network related to LRI

In the adjusted univariable analyses, every additional 10% of the network living more distant, who were 5 to 15 years younger and friends were associated with a 7, 9 and 8% higher odds of reporting LRI, respectively. Every additional 10% of the network of the same age or family members were associated with a 8% lower odds of reporting LRI (Table 3).

In the multivariable detrimental exposure model, every additional SD in age heterogeneity and every additional 10% of the network that were friends and who were 5 to 15 years older and 5 to 15 years younger were associated with a 3, 12, 12 and 11% lower odds of reporting LRI, respectively. In the multivariable beneficial exposure model, every additional 10 contacts were associated with a 1% lower odds of reporting LRI. Moreover, with every additional 10% of the network that were alters of the same age or family members the odds of reporting LRI was 11 and 9% lower, respectively (Table 4).

### Social network related to GI

In the adjusted univariable analyses, every additional alter reported was associated with a 3% higher odds of reporting GI. With every additional 10% of the network that were alters with a moderate contact frequency (quarterly), who lived further away, who were 5 o 15 years younger, or friends, the odds of reporting GI was 10, 7, 9 and 12% higher, respectively. With every 10% increment of the network that were alters contacted on a daily or weekly basis, household members, and family members the odds of reporting GI was 5, 13, and 9% lower, respectively. Having a partner was associated with a 26% lower odds of reporting GI (Table 3).

In the multivariable detrimental exposure model, the part of the network that were friends remained independently associated with GI. In the multivariable beneficial exposure model, every additional 10% of the network of alters of the same age, family members, and acquaintances was associated with a 7, 11, and 9% lower odds of reporting GI, respectively (Table 4).

### Social network related to UTI

In the adjusted univariable analyses, no statistically significant associations were found between network parameters and UTI (Table 3). In the multivariable detrimental exposure model, every additional 10 contacts were associated with a 2% higher odds of reporting UTI. The multivariable beneficial exposure model revealed no statistically significant associations (Table 4).

## Discussion

The current study is unique, as we were able to combine sophisticated, real-time social network data with infection prevalences in an epidemiological study on four different infectious diseases: URI, LRI, GI and UTI. We identified both detrimental and beneficial associations of social network parameters with the prevalence of infections. We observed that larger network size was associated with a higher prevalence of URI and GI, while a high total number of contacts was associated with less URI and LRI. In addition, participants with networks mainly composed of friends presented a higher prevalence of LRI and GI, as opposed to those with a higher family percentage, who presented lower LRI and GI prevalences. Finally, a higher percentage of network members of the same age was associated with lower URI, LRI and GI prevalences. We found no clear associations with UTI, although a high total number of contacts was associated with higher UTI prevalence.

In the present study, social network size was associated with a higher prevalence of URI and GI. A likely explanation for our findings is that a larger network indicates exposure to a greater range of infectious agents, and therefore leads to a greater incidence of symptomatic infections. In addition, the likelihood of meeting an infected person is higher in a large network. We observed no association between network size and UTI. This is in line with infection spread theory, i.e. that infections that are transmitted through the air or direct contact (URI, LRI and GI) are spread through social networks, whereas UTIs, mainly caused by commensal bacteria, are not. UTI however was positively associated with the total number of contacts. The underlying mechanism that explains how more contacts, independent of social network size, is related to an increased risk of UTI remains unclear in the present study, and may be a subject of interest in future research.

A higher total number of contacts was associated with lower URI and LRI prevalences. This association was independent of network size. This is in line with a previous study, which showed that susceptibility to URI was lower among participants with more types of social ties [9, 13]. Our results indicate that network size and total number of contacts have an independent association in opposite direction with infection prevalences. Hence infection prevalence is reflected by both of these measures, and not merely as a function of increased infection spread in relation to the network, and therefore both should be considered in studies on their association with infections.

Independent of the number of alters and contacts, participants with networks composed of a relatively large percentage of friends presented a higher prevalence of LRI and GI. Contacts with friends are assumed to be intimate, including touching or kissing, and play an important role in the transmission of LRI and GI. This is in line with previous research, which suggests that friends are a group with a high potential for transmission [30], and in line with a study that proposes monitoring the friends of randomly selected individuals as a novel strategy for early detection of influenza [51]. Note that when a person’s network contained a relatively high percentage of acquaintances, the prevalence of GI was lower. A possible explanation for the latter is that GI transmission requires surface contamination or physically direct contact, and contacts with acquaintances (neighbors or colleagues) tend to be less close than contacts with friends. A large percentage of family members within the network was associated with fewer reported LRIs and GIs. Previous research has shown that the family is an important source of social support [52], and higher levels of social support have been shown to enhance several aspects of immune function [11, 12]. The ego’s family may act as a buffer for LRI and GI through high levels of social support, indicating a positive impact on lower susceptibility to these infections.

Notably, while some mathematical models consider the number of household contacts to be highly relevant for disease transmission [21, 22, 28, 30, 31, 33], we did not find such association. On the contrary, the present results show that a relatively large percentage of household members is associated with lower URI and GI prevalences.

A high percentage of alters in the same age range (± 5 years) was associated with lower URI, LRI and GI, but not with lower UTI. Mathematical models usually incorporate age-mixing patterns, which range from fully assortative (individuals infect only those in the same age group) to disassortative (those of one age group only infect individuals in another age group) [16, 22, 28, 33]. Our current study confirms that individuals tend to mix assortatively (44% of participants had contacts with others within the same age group) [22, 28, 33]. However, while mathematical models usually assume that individuals infect those in the same age group, we found the opposite effect: when the network contains a relatively high percentage of alters of the participant’s age, lower URI, LRI and GI prevalence was observed. A high variability in ages of alters (age heterogeneity), as well as a high percentage in alters that were younger or older (5 to 15 years) was associated with higher LRI prevalence, which may indicate exposure to a wider range of infectious agents from people in broader age ranges, and as transmission rates were higher among children and the elderly [53], higher variability in age and dissasortative mixing patterns may indicate more contacts with high-transmission risk individuals. To the best of our knowledge, this is the first study that assessed the associations between mixing or heterogeneity within the composition of a network and the prevalence of symptomatic infections, revealing new insights into the transmission potential of assortative and disassortative age mixing patterns.

One strength of the present study is that it provides new empirical data on the social network at the participant level. This has given us the opportunity to examine a set of questions that, to the best of our knowledge, have not been addressed before. Recent research on infection dynamics using mathematical models shows the importance of contact patterns for transmission dynamics and the use of parameters estimated directly from contact data. Our study improves the understanding of the epidemiology of infectious disease, and can be used to complement mathematical models of infection spread on the importance of network composition for the estimation of transmission parameters, as well as in further epidemiological research on the association of specific network parameters and the prevalence of several infections.

Nevertheless, this study also has limitations. First, as the data was cross-sectional, causal relationships could not be examined. Second, self-reporting may be subject to bias. Although the self-reporting of infections has been used successfully in the past in relation to network assessment [23, 28], symptoms may be under or over-reported. Third, as contact networks are, by definition, hard to measure [14] our assessment and computed network parameters also have strengths and limitations. Social networks can be measured by different methods, such as reviewed by Read et al. [17], one of which is the egocentric approach used in the present study. Egocentric networks are useful for measuring likely proxies of the true underlying network of potentially infectious contacts, yielding valid data and insight in ego-network composition in relation to infections [13, 22, 33, 54]. Fourth, the assumptions made to calculate the total number of contacts may under- or overestimate the actual total number of contacts. For every alter named, the highest contact frequency (e.g. monthly contact) was used as an indicator of actual contact frequency, and as participants report to meet one alter every month on several questions, this alter is assumed to be met on a monthly basis. It is possible however that the participant met this alter once a month for one activity, but also once a month for another activity. Moreover, the “daily or weekly” answer category was assumed to refer to two contacts per week. This assumption, too, may result in an underestimation of the actual contact frequency.

Moreover, the questionnaire consisted of seven questions on different types of interactions, some of these types require direct close proximity interactions (such as visits for social purposes or offering practical help), and other types of interactions may also have occurred by telephone/internet conversations (such as provision of emotional support or advise on problems). Therefore, estimations of the total network size and total contact frequency may over represent the actual number of close proximity interactions, as a maximum of 13–14% of the interactions may potentially not have occurred in close proximity.

Another limitation of the questionnaire was missing information on the duration of contacts between the ego and the alters, which has been shown to be highly relevant for disease modelling [25, 26]. Additionally, use of public transport has not been measured, which may have impact on the number of contacts participants made in the study period. However, previous research has shown that the transmission rate in public transport is low [55].

Finally, The Maastricht Study has a population based design and is enriched with type 2 diabetes participants for reasons of efficiency; i.e. to increase the statistical power to identify any potential contrasts between individuals with and without type 2 diabetes. Subsequently, participants were slightly more overweight/obese than the general population [56]. To control for this design-related oversampling, we adjusted all analyses for diabetes status and BMI. Although higher prevalences of type 2 diabetes and overweight may have slightly increased the prevalences of infections within the study population, odds ratios are likely unbiased. Another limitation of the design is that the study population consisted of adult participants only and it is unknown whether results are representative for people younger than 40 years of age. Further, the age range of the participants probably leads to an underrepresentation of children in the ego’s network, while it has been shown that children and teenagers may have an important role in the spread of close-contact infections [33].

Moreover, the extensive phenotyping forces participants to cover 4 half-day visits at the research center, which may be difficult for people who work and travel a lot and have many contacts. To enable participation for those more “busy” people, visits were also offered in the evening hours and on Saturday. The design of the present study may have some limitations, but it is also unique in the examination of the association between social network parameters and infections, and may therefore lead to new insights in the understanding of the epidemiology of infectious diseases.

## Conclusions

To conclude, social network size and total number of contacts were important determinants for the prevalence of URI, LRI and GI. Moreover, the composition of the social network in terms of types of alters (friends, family, age) appears to be related to the risk of infection. While further studies are needed to examine underlying mechanisms and causality, our findings could have important implications for the estimation of transmission parameters to optimize current infectious disease control, and can be used for the development of non-pharmaceutical infection prevention strategies.

## Additional files

Additional file 1: Statistical formula for the derivation of the Index of Qualitative Variation. Statistical formula for the derivation of the Index of Qualitative Variation, with an example how to compute the IQV. (DOCX 19 kb)

Additional file 2: Table 1. Associations between characteristics of the participants and self reported upper- and lower respiratory, gastrointestinal, and urinary tract infections in The Maastricht Study (*n* = 3004). The associations of demographic characteristics (sex, age, type 2 diabetes, body mass index, season, educational level, smoking status, alcohol consumption, and employment status) with URI, LRI, GI and UTI were shown. (DOCX 21 kb)

## Abbreviations

GI: gastrointestinal tract infection
LRI: lower respiratory tract infection
URI: Upper respiratory tract infection
UTI: urinary tract infection
