# Supplementary material for: Social networks in relation to self-reported symptomatic infections in individuals aged 40–75 - the Maastricht study –
Source: BMC Infect Dis. 2018 Jul 4;18:300. doi: 10.1186/s12879-018-3197-3 (PMC6030801; doi:10.1186/s12879-018-3197-3)
Supplement: Supplementary file 1 — Statistical formula for the derivation of the Index of Qualitative Variation. Statistical formula for the derivation of the Index of Qualitative Variation, with an example how to compute the IQV. (DOCX 19 kb) [file 12879_2018_3197_MOESM1_ESM.docx]

**Additional File 1: Statistical formula for the derivation of the Index of Qualitative Variation**

To assess sex heterogeneity within the ego’s network, we computed the Index of Qualitative Variation (IQV) by Mueller and Schuessler (1961) [46]. The IQV is defined as the ratio of observed differences divided by maximum possible differences, where “0” represents a fully homogeneous and “1” a fully heterogeneous network [46].

Observed differences were calculated as

$$Observed differences=\sum f_{i}f_{j} i\neq j$$

Where f refers to the frequencies of category i,j.

To calculate the maximum number of possible differences (MPD), we used the formula

$$\mathrm{MPD}=\frac{c \left( c-1 \right)}{2} \left( \frac{n}{c} \right)^{2}$$

Where c is the number of categories, and n is the number of observations.

The IQV was computed as

$$IQV= \frac{observed differences}{maximum possible differences}$$

Below we show the derivation of the IQV for an ego network with 7 men and 4 women.

The observed differences were

$$Observed differences=7*4=28$$

In a network of 11 network members, the maximum possible differences were

$$\mathrm{MPD}=\frac{2 \left( 2-1 \right)}{2} \left( \frac{11}{2} \right)^{2}=\left( \frac{11}{2} \right)^{2}=30.25$$

In a network of 11 members, of which 7 men and 4 women, the IQV is

$$IQV= \frac{28}{30.25}=0.926$$
