# Supplementary material for: Social networks in relation to self-reported symptomatic infections in individuals aged 40–75 - the Maastricht study –
Source: BMC Infect Dis. 2018 Jul 4;18:300. doi: 10.1186/s12879-018-3197-3 (PMC6030801; doi:10.1186/s12879-018-3197-3)
Supplement: Supplementary file 2 — Table 1. Associations between characteristics of the participants and self reported upper- and lower respiratory, gastrointestinal, and urinary tract infections in The Maastricht Study (n = 3004). The associations of demographic characteristics (sex, age, type 2 diabetes, body mass index, season, educational level, smoking status, alcohol consumption, and employment status) with URI, LRI, GI and UTI were shown. (DOCX 21 kb) [file 12879_2018_3197_MOESM2_ESM.docx]

**Additional Table 1. Associations between characteristics of the participants and self reported upper- and lower respiratory, gastrointestinal, and urinary tract infections in The Maastricht Study (n=3004).**

|  | **Upper respiratory tract infection** | | **Lower respiratory tract infection** | | **Gastrointestinal tract infection** | | **Urinary tract infection** | |
| --- | --- | --- | --- | --- | --- | --- | --- | --- |
|  | **OR** | **95% CI** | **OR** | **95% CI** | **OR** | **95% CI** | **OR** | **95% CI** |
| **Sex (male)** | 0.94 | 0.79-1.11 | 0.94 | 0.74-1.21 | 0.86 | 0.68-1.09 | **0.43***** | **0.29-0.61** |
| **Age (years)** | 0.99 | 0.98-1.00 | 0.98^#^ | 0.97-1.00 | 0.98^#^ | 0.97-1.00 | 1.00 | 0.98-1.03 |
| **Type 2 diabetes (yes)** | 1.01 | 0.82-1.24 | 0.97 | 0.72-1.31 | **1.60**** | **1.22-2.09** | **2.05***** | **1.38-3.05** |
| **Body Mass Index (kg/m²)** | 1.01 | 0.99-1.03 | **1.05***** | **1.02-1.08** | 1.02 | 1.00-1.05 | 0.99 | 0.96-1.03 |
| **Season (winter)** | **2.29***** | **1.95-2.70** | **2.43***** | **1.91-3.10** | 1.07 | 0.86-1.34 | 1.17 | 0.85-1.62 |
| **Educational level** |  |  |  |  |  |  |  |  |
| - low | 1.00 |  | 1.00 |  | 1.00 |  | 1.00 |  |
| - medium | 1.07 | 0.86-1.33 | 1.28 | 0.94-1.74 | 1.20 | 0.89-1.62 | 0.86 | 0.56-1.31 |
| - high | **1.28*** | **1.04-1.57** | 1.11 | 0.82-1.51 | **1.37*** | **1.03-1.83** | 0.99 | 0.66-1.48 |
| **Smoking status** |  |  |  |  |  |  |  |  |
| - never | 1.00 |  | 1.00 |  | 1.00 |  | 1.00 |  |
| - former | 1.08 | 0.90-1.29 | 1.03 | 0.79-1.34 | 1.07 | 0.83-1.37 | 0.90 | 0.62-1.31 |
| - current | **1.44**** | **1.11-1.86** | 1.36^#^ | 0.95-1.94 | 1.20 | 0.85-1.71 | **1.78*** | **1.12-2.81** |
| **Alcohol consumption** |  |  |  |  |  |  |  |  |
| - Non-consumers | 1.00 |  |  |  | 1.00 |  | 1.00 |  |
| - Low consumers | 0.89 | 0.71-1.11 | 0.92 | 0.67-1.26 | 1.13 | 0.82-1.55 | 0.95 | 0.62-1.45 |
| - High consumers | **0.76*** | **0.59-0.99** | 0.81 | 0.55-1.18 | 1.30 | 0.91-1.86 | 1.11 | 0.68-1.80 |
| **Employment status** |  |  |  |  |  |  |  |  |
| - Employed | 1.00 |  | 1.00 |  | 1.00 |  | 1.00 |  |
| - Unemployed/ retired | 1.20 | 0.91-1.59 | 1.40^#^ | 0.95-2.06 | 0.97 | 0.65-1.44 | 1.06 | 0.63-1.80 |
| - Not known | 0.92 | 0.74-1.29 | 1.13 | 0.84-1.53 | 0.91 | 0.68-1.21 | 0.75 | 0.49-1.15 |

OR Odds Ratio, 95%CI; 95% Confidence Interval, # p<0.1, *p<0.05, **p<0.01, ***p<0.001 .

All analyses were adjusted for: sex, age, smoking status, diabetes status, alcohol consumption, educational level, employment status and season.
